# Saudi Arabian Students’ Beliefs about and Barriers to Online Education during the COVID-19 Pandemic

**DOI:** 10.3390/children9081170

**Published:** 2022-08-04

**Authors:** Mohaned G. Abed, Reem F. Abdulbaqi, Todd K. Shackelford

**Affiliations:** 1 Department of Special Education, Faculty of Educational Graduate Studies, King Abdulaziz University, Jeddah 21589, Saudi Arabia; 2 Department of Sociology and Social Work, Faculty of Arts and Humanities, King Abdulaziz University, Jeddah 21589, Saudi Arabia; 3 Department of Psychology, Oakland University, Rochester, MI 48309, USA

**Keywords:** COVID-19, higher education, online learning, attitudes, university students, Saudi Arabia

## Abstract

At a time when pandemics such as the novel coronavirus (COVID-19) spread rapidly, the deployment of online education is essential. However, to successfully leverage online education in such times, it is important to investigate learners’ motivations and beliefs about online education and associated barriers as well as the role of religious and social values. To investigate these motivations and beliefs, this study included semi-structured interviews with 10 female undergraduate students. These interviews explored the perceptions of students with regard to their engagement with online learning and assessment amid the COVID-19 pandemic in Saudi Arabia’s higher education system. The findings indicate that the challenges linked to the sudden shift in learning mode and changes in assessment techniques impacted students’ engagement with learning and assessment. The findings also indicate that personal challenges decreased the willingness of students to learn online, but that their beliefs about learning online were improved by the quality of online learning. The study identified that one of the most important elements of improving beliefs about online learning is open communication between students and instructors, as this contributes to shared understanding and acceptance. The results are presented and discussed in connection with current literature, research implications, and future directions.

## 1. Introduction

Studies addressing the effects of COVID-19 on higher education have been conducted primarily in the Western world [1,2]. Notwithstanding the fact that a few global studies have been conducted with regard to how COVID-19 has affected education, the focus and implications of these studies are directed towards developed countries, with little attention being paid to the developing world, such as countries in the Middle East, including Saudi Arabia [3].

The shift to online learning was announced by the Saudi Arabian government on 8 March 2020, with the change going into effect the next day in all Saudi Arabian colleges, universities, and K-12 schools. For universities that had already implemented online learning using Blackboard, among other learning platforms, this announcement accelerated these changes. The shift occurred in the second mid-term week for universities. Teaching shifted abruptly and completely to online delivery, producing many problems such as unfinished examinations [4].

Saudi Arabia was one of the first Middle Eastern countries to implement complete quarantine and, therefore, the urgent transition to online education. After the lockdown of universities in Saudi Arabia during the COVID-19 pandemic, and to sustain the education process continuously, the Saudi Ministry of Education developed an alternative policy to facilitate online education [5]. The Ministry of Education had already provided resources to public universities to support virtual classrooms and online learning [6]. Presently, all universities in Saudi Arabia utilise the Blackboard platform, which is one of the most frequently used online platforms. This made the experience of online education more flexible and able to address obstacles to communication between instructors and their students [7,8].

## 2. Literature Review

The impact of the COVID-19 pandemic on policymakers and universities in Vietnam was addressed by Pham and Ho [9]. These scholars emphasised the mounting appreciation of the virtues of e-learning and other linked technology-based educational modalities. In the context of Saudi Arabia, Alateeq et al. [10] focused on the mental health of Saudi Arabian students during COVID-19. These scholars concluded that there was a greater impact on university students than on intermediate and secondary school learners. Some of the sources of stress among students in universities were linked to loneliness, parental matters, curricula, and apprehension regarding performance and failure in examinations.

Research investigating the deployment of online education indicates a correlation between direct teaching and successful learning [11]. As online education has progressed, learners are now viewed as recipients of knowledge and as active participant constructors of knowledge. The purpose of technology has shifted from providing drill and practice (i.e., controlled learning) to the successful use of new tools and a creative environment for learners to solve everyday problems, which facilitates learning [12]. Online education has expanded alongside the widespread adoption of internet technology. Researchers such as Jagger [13] have noted that aspects of the delivery of online coursework can positively affect learner beliefs about and successful participation in online education. Therefore, these researchers have sought to identify the tools for effective delivery of online education and have argued that learners’ non-academic and technical skills play an important role in their successful participation in and completion of online courses [14].

According to Grundmann et al. [11], the absence of in-person laboratory and hands-on experiences in online education is a significant challenge for some learners, especially for courses in technical and applied fields. However, Grundmann et al. [11] provided evidence that the format of delivery—online or traditional (i.e., in-person)—does not significantly impact the achievement of learning outcomes for science-related courses.

In recent years, researchers have hypothesised that instructor-related variables impact the effectiveness of online course delivery more than learner-related variables. These researchers have argued that student success in achieving learning outcomes depends less on the course format (i.e., in-person vs. online) and more on the use of appropriate pedagogy, quality of instruction, and effective instructor–learner interaction [15]. Online education researchers have reported that instructor–learner communication is important for learners’ success, given the nature of online courses. They suggest that effective communication between learners and instructors must be cultivated to motivate students and to facilitate cognitive engagement in online environments [16]. Given the literature indicating the importance of learner beliefs about and engagement with online learning, it is important to identify specific learner beliefs about online education and then to develop pedagogy to foster positive online educational environments that facilitate successful learning. A number of previous studies have concluded that learner beliefs play an important role in the process of learning [17]. Such conclusions have inspired several scholars to investigate the beliefs of learners about online learning and other learning facilitated by technology.

The previous literature has focused attention on the challenges and difficulties that learners face in classes conducted online. Some of the challenges include the availability of online technical support and assistance. These studies indicate that without technical support during online learning, students may be anxious, a situation that could have a negative impact on learning [18].

Bloomfield and Jones [19] explored the beliefs of first-year students about online learning courses. The same researchers also investigated the practices of the students during the course used to supplement traditional methods. The researchers concluded that even though the beliefs of students toward online learning were positive, they preferred to attend traditional classes. On the other hand, many of the students felt frustrated because of several issues, including technical difficulties, navigational issues, and underestimating time requirements.

In the wake of technological innovations, educators have been afforded opportunities to enter new learning environments, including online environments. Nevertheless, many adult learners prefer conventional in-person learning environments. The present study examined Saudi Arabian university learners’ perceptions of and beliefs about online education during the COVID-19 pandemic. The present study is situated within the literature addressing online learning pedagogy to offer insights to instructors required to transition in-person courses to online courses during the COVID-19 pandemic.

Online learning has expanded in the wake of the COVID-19 pandemic. However, a barrier to this transition is decreased student retention [20]. Many learners and instructors remain sceptical about online course delivery. There is a need to identify the challenges learners associate with online learning and a need to identify what can be done to address these challenges, including establishing pedagogical models that afford successful deployment of online education for both learners and instructors. The present study is the first step toward a more successful deployment of online education. We intended to identify differences between learners who are more positively disposed to online education and those who are less positively disposed to online education and to offer tentative recommendations to instructors for maximising the successful delivery of online courses.

This study intended to address the following research questions:

1. What barriers might learners face when taking online courses?
2. What differences might exist between learners who are more positively disposed to online learning and those who are less positively disposed to online learning?
3. Our unique sample of Saudi Arabian students affords the opportunity to address the question: What might be the impact of Islamic values and society on online learning?

The present study is important because it explored a relatively novel teaching and learning experience. Identifying the relevant perceptions and beliefs of some of the most important stakeholders, particularly students, could assist in improving the way higher education is delivered in Saudi Arabia. The present study used a relatively unique method of initially investigating and tentatively identifying the immediate impacts and long-term effects of a sudden shift to online learning on the psychological, social, and academic wellbeing of Saudi Arabian students. The study also examined how students perceive and react to the success or failure of the online teaching and learning experience.

## 3. Methods

### 3.1. Study Design

This study used a phenomenological approach as a qualitative method to explore students’ beliefs about online learning during the COVID-19 pandemic, with a focus on identifying beliefs about and barriers to online education and the potential impacts of religious and social values on online education. A phenomenological approach describes “the common meaning of several individuals of their lived experience of a concept or a phenomenon” [21]. Semi-structured interviews were conducted to afford a qualitative analysis. The qualitative method can generate an in-depth initial understanding through assessing participants’ lived experiences, and may identify potentially productive avenues for future research with larger samples that can afford quantitative analyses [21].

### 3.2. Participation and Procedure

Saudi students attending one of several public universities were invited to participate in the study. Regarding inclusion criteria, all participants were Saudi undergraduate students taking classes online during the interviews, and had experienced the sudden shift from in-person to online education coinciding with the COVID-19 pandemic. Participants were aged 19 years or older and had to be current public university students.

We were offered a limited window of time to collect data for the current research. A convenience sample of 10 female students was recruited in several ways. Emails were sent to students inviting them to participate. Communication occurred through social media. Snowball sampling was also utilised. This means that students who participated in the study were asked if they could refer another student to participate. After participants had been vetted and provided informed consent, they were interviewed. Each interview lasted between 40 and 50 min. We recognise that our sample is small and likely biased in terms of student availability and interest in participating in this research, notably during a difficult transition from in-person to online coursework coinciding with the announcement of the global COVID-19 pandemic. Although we contacted male and female students, only female students responded and participated. The female students that participated were the first to respond. Unfortunately, we do not have information on why they responded when they did respond, nor do we have information on why no males responded.

Because of the difficulties of in-person interviews, since there was a need to observe social distancing, phone and zoom interviews were conducted. The interviews occurred in March 2021. Table 1 presents available demographic information for the 10 interviewees.

### 3.3. Ethical Considerations

The study was conducted in accordance with the Declaration of Helsinki, and approved by the Research Ethics Committee at King Abdulaziz University (protocol code: IFPHI-378-324-2020; date of approval: 4 June 2020).

Verbal consent was obtained at the beginning of each interview. All participants were reminded that participation is optional and that they could withdraw without penalty at any time. Participants were assured that all data would only be used for research purposes, and their identities would be concealed using codes.

The research objectives were stated at the beginning of the interview and before securing participants’ consent. At the end of each interview, participants were walked through all the information gathered so that they could correct any misunderstandings.

### 3.4. Data Collection

In this study, in-depth interviews were semi-structured with open-ended questions, affording participants the freedom to control pacing and interview subject matter [21]. Additional sub-questions were asked as needed based on two kinds of information probes in which the researcher encouraged the interviewee to talk more about the current question. The other sub-questions were based on markers at which the interviewer noted an important point in a previous answer and requested additional, often clarifying, information [22]. All interviews were conducted in the participants’ native language (Arabic), and most of the participants used an informal dialect of Arabic that the interviewer was familiar with.

### 3.5. Interview Protocol

The interview questions were 10 open-ended questions that began with asking the participant about her major, followed by more in-depth questions about her educational experience. The questions moved from questions about the participant’s experience with and perceptions of online learning before the pandemic to their lived experience throughout the pandemic. Participants also were asked about their personal views about online leaning during the pandemic and the associated changes that had occurred in their academic life. Other questions addressed the various challenges and difficulties participants may have faced, such as organisational, economical, technical, and social challenges. Finally, participants were asked about their social and religious values in relation to their online learning during the pandemic. Interview questions were generated by the researchers based on a comprehensive review of the literature.

The interview started with the interviewer developing and establishing rapport with the interviewee, followed by a preview of the study and obtaining consent to participate from the participant. Key questions were asked following an interview guide developed for this study and which two academic professors subject matter experts reviewed to ensure that the questions were clear and concise. All questions are referenced in the presentation and discussion of the results (below). At the end of each interview, participants were thanked for their participation and were asked if they could refer others to participate.

### 3.6. Data Analysis

All interviews were audio-recorded, and all recordings were transcribed in Arabic. A colleague familiar with the informal Arabic dialect examined the scripts by comparing them with the original recordings. The Arabic script was used in the data analysis process. Major quotations were translated into English to be used in presenting the results (below). To achieve accuracy in the quotations presented, a re-translation to Arabic was performed by a qualified translator to ensure that the meaning remained the same.

For the data analysis, a thematic approach was used. This approach involves first reading and transcribing the responses collected from the interviews to get a general sense of the whole and the ideas presented. This was followed by extracting, from each script, significant statements and phrases relating to the phenomena of interest to the current study. After that, meanings were formed from the significant statements, and these were regarded as themes. The themes were developed into clusters, and then into categories. A colour-coded system was used to highlight specific themes and categories to perform an initial analysis. As a result, the researchers were able to generate a description of the lived experiences of participants and to identify similarities and differences across participants. The procedure used in the present study required continual comparison between the data and the emerging analysis. The analysis and the meanings were considered with reference to the Saudi context (i.e., Islamic teachings and distinct social aspects of Saudi culture).

## 4. Results

Based on the in-depth interviews, four themes emerged from the analysis:

- Beliefs about online learning;
- Motivations for taking online classes;
- Barriers to online learning;
- Islamic and societal norms of online learning.

### 4.1. Beliefs about Online Learning

Taking online classes during the COVID-19 era was mandatory across Saudi Arabian universities. This is an aspect that all participants referred to at the beginning of each interview. Perhaps due to this mandatory nature, students expressed their feelings about online learning as “not bad” and “I can get used to it, but with limitations”, whereas others said that “I cannot wait to go back to the face-to-face setting”, and one stated, “I was in denial [about the possibility of moving to online learning] until it happened”.

Rana, a fourth-year humanities student, stated, “Although online classes give me more time, I learn better in a face-to-face setting. Learning online is not bad, but if I had the choice, I would choose face-to-face”. Another student, Sarah, also a fourth-year humanities student, said, “I enjoy taking online classes because it is easy to attend, but it does not suit the applied classes (such as field placement) which I need for my major. Therefore, I support online learning for classes that do not need application.” Even though some students, such as Sarah, said that online classes are more suitable for theoretical classes, other students did not agree. Hesah, a second-year humanities student, said, “I miss face-to-face debates where I can see face and body expressions that enrich the learning experience”.

Some students identified online learning as a positive change because it gave them more time during the day. Hanan said, “Online classes gave me more time because I live on the north side of the city--Jeddah. I usually take about an hour or so to get to the university without traffic which will add about another hour”. Because of this, some students considered online learning a preferable way of learning. As Rahma said, “I think this is a new way of learning, and I feel it will continue in the future. I might choose to study in an online program in the future”.

From the participants’ point of view, they took online classes for the first time with limited experience. The sudden change may have affected how they perceived online learning. Most participants perceived online education as a mandatory way of learning, not as a different way of learning. Even though they admitted that they use different ways of attending classes and doing homework as a new experience, they still preferred a face-to-face setting.

No participant clearly stated that they are opposed to online learning. Some of the participants pointed out that they are committed to taking the online classes, and they are doing their best to keep up with what they are missing, for example, by watching and re-watching recorded lectures.

### 4.2. Motivation for Online Classes

When asked about what motivated them to take online classes, most of the participants answered, “It is the only way to progress in my study” or “to graduate on time”. They noted that transitioning to online classes had not been easy, and they had to make significant adjustments to their routine. One of the participants, Hesah, said, “I struggled to find a way to be focused. I live in a small apartment with my family and four younger brothers, and I can hardly focus on my studies. Before online classes, I usually spent my time at the university where I can find quiet and the space to focus on studying”.

Furthermore, Hanan said, “The only thing that motivates me to take online classes is that I am studying what I like, and I like my major, so whatever way I need to do it, I will do it, and now it is online”. On the other hand, Nada said, “Online classes pose a challenge for me. It is very easy to miss a class due to falling asleep in front of the laptop. Or sometimes you get pressure from your family to spend more time with them, and you can watch the recorded lecture later. Sometimes it could affect your grades because it is mandatory to attend classes live for attendance points”. Sarah said, “I wanted to graduate on time. It was my only goal; I started to learn about the program that we would use as soon as they announced that classes would be online. It was difficult for me to find the lecture and connect to the class initially, but after a while, I got used to it”.

However, some participants felt that online learning gave them more time to manage their day by reducing the hours they spent in transit to and from the university, as stated in the previous theme. This worked as motivation for the participants, such as Noor, who said, “I consider online learning as similar to regular learning; the only differences are the place and the method. I got to have more time for studying”.

Students attending public universities in Saudi Arabia do not pay tuition. That said, it is possible they feel that they do not have the entitlement to ask for a particular method of learning or that they have the right to withdraw or postpone their academic year until face-to-face learning resumes. Some universities offered students the opportunity to withdraw from a semester or a class during the first transition period, but none of the participants took that opportunity. They felt that they did not need it, and most of their professors were considerate about the situation.

To motivate students to take online classes, educational institutions might adopt different ways to incorporate the use of technology into traditional classes, such as hybrid classes that allow students to attend either online or in-person (when possible). This could help reduce the resistance to taking an online class and increase motivation to take an online class.

### 4.3. Barriers to Attending Online Classes

Participants were asked if they faced barriers or difficulties while taking online classes. Three types of barriers emerged from their responses: technological barriers, economic barriers, and educational barriers.

Regarding the technological barriers, internet connection difficulties were mentioned by most of the respondents. Rahma said, “Sometimes the program gets jammed, and I think that is because of the huge numbers of students who are using the program at the same time”. Additionally, Nada said, “The internet connection in my house is weak, so sometimes when I upload homework or take an online exam, the internet gets lagged and closes the browser without going through the submissions, which cost me my grades”. Additionally, this was confirmed by several other participants. Furthermore, Hanan said, “Some professors do not record the lecture, so if I could not attend the class for technical reasons, I cannot go back and listen to the recording later”.

Another technological issue was related to cancelled classes due to technical issues. Some participants noted that it was difficult to deal with cancelled classes because they needed to compensate for any material that might have been missed. Jana said, “Sometimes the program gets lagged, and the class gets cancelled because of it, so we had to take a compensation class at another time, which will usually be late at night or the weekend, and attendance was mandatory”. These student views show that the application of online education needs continual support to solve arising issues that may generate technological barriers. Some of these barriers are manageable, such as the timing or the recording of the lecture. Other barriers are difficult to manage, such as the strength and stability of an internet connection, because these depend on multiple factors, for instance, the type of device, area of residence, signal availability, and program capacity.

The second barrier that emerged from the responses was economic barriers, especially the cost of devices and internet access. Participants explained that switching to online learning came at a high cost to them and their fellow students. Nada said, “At the beginning of online classes, I used prepaid data to get access to the internet, but that did not support the heavy load that the program needs to run. I had to ask my family to buy an internet plan, so I would not have problems connecting to the classes”. Another participant, Rana, said, “I used to go to the university and use the computer at the library since my old laptop got broken and my family cannot afford a new one. At the beginning of online classes, I used my old phone to get to classes, but I had to buy an internet card to recharge my phone. After a while, some good people gave my dad a used laptop and a Wi-Fi device so my siblings and I could share using them for studying and attending classes”.

Although some participants noted that they did not face economic difficulties, they said that many of their friends were affected. Hanan said, “My poor friends always need to recharge their mobiles with an internet card to attend classes, and most of the time, it failed”. Additionally, Noor said, “Online education could be costly for those with multiple kids, and each one of them needs to be in class at the same time. That means that the family must provide a device for each kid”.

The third barrier was educational issues related to the way classes and homework are managed. Some participants stated that some professors were inconsiderate about the burden that students carried due to the sudden switch to online classes. Noor said, “Some professors reduced the number of exam questions and put high marks on each question, so just because I did not answer three questions well, I end up losing six marks or more. Other professors believe in electronic grading and refuse to check if there were mistakes in the grading”. Another participant, Sarah, said, “Exam time is critical because if the exam was ended due to the internet connection, we have to contact the professor immediately so they can reopen the exam. Some of the professors were cooperative, and others were not”. Hence, Hesah said, “Some professors don’t have mercy; if you did not submit on time or did not attend a class, they immediately deduct from your grades”.

One of the participants complained about the amount of homework assigned to them during the online classes. Sarah said, “The professors took advantage of us being at home and started to assign two or three homework assignments a week, and their justification is that we are home and have nothing to do”. Other participants complained about the way some professors conducted their classes. Hesah explained, “Some professors found it hard to deliver information online. One professor talks and talks and I get lost and can’t concentrate”.

Students at the time of the pandemic had to deal with major changes in education and other features of life. Social distancing and not being able to follow their usual routine may have affected the mental, physical, and social aspects of their lives. To deal with these multiple challenges, students should be treated in a holistic way and not only from the academic or educational angle. This implies that when professors work with students, they should be considerate about what the students face and what challenges they are enduring.

### 4.4. Social and Religious Values

Some of the social aspects that emerged from the data were related to social support and family bonding. As Rana previously said, some people helped provide a laptop and internet for her and her siblings to attend their classes. Other participants noted that there is some governmental help, such as social security services, which offer assistance to those who cannot afford online education essentials.

As for family bonding, some participants shared how being at home more brought them closer to their families. Sahar said, “My mom is happier now that I am always at home”. On the other hand, some participants felt that being at home brought new challenges. Rana said, “Our house is so small, and the situation was as if we are having a family gathering all the time. My siblings run around screaming, and they wake up very early. I cannot participate orally and always write my responses during class”. Another participant, Jana, said, “My mom got tired of hearing me saying I have class, or I have work to do. With the amount of studying that I had, I could barely sit with my family”.

Some of the religious, ethical, or moral values that the participants talked about were fairness, collaboration, honesty, and truthfulness. Noor stated, “Some professors are fair when dealing with a different student in different situations”. Additionally, Sarah said, “To be sincere, some professors were very supportive and cooperative with us; even when they assign homework, they leave it open in terms of deadlines”. Hence, Hesah said, “Students also collaborate to perform better in their education”.

One of the values was a challenge for some participants: honesty in not cheating on exams. Sarah explains, “In the time of online studying, it’s not easy to maintain honesty. You have the books and the notes in front of you, so it is up to you to resist opening them. Therefore, self-censorship is the key”. Nada agrees, saying, “I remember that I could open a book during one of the exams, but my consciousness and my Islamic values kept me from not doing so”. All participants agreed with this sentiment.

## 5. Discussion

That the COVID-19 pandemic transformed the world and prompted countries to take drastic measures such as lockdowns is well-established. COVID-19 also impacted the education system. The rapid changes implemented as a response to COVID-19 took many stakeholders by surprise as they transformed the delivery of education drastically. It is posited that as a result of unequal access to the internet, some students experienced various challenges when attempting to attend online lectures, which resulted in stress for some students more than others. Other students came to realise that their homes were not suitable for learning because of comfort and family factors. Many students indicated that they were apprehensive about their academic performance because they were not psychologically prepared for the changes that were introduced.

The data collected in this study shows that the beliefs of Saudi Arabian university students regarding the online classroom are generally positive, at least in this small convenience sample of female students. This is a view that is supported by several scholars, e.g., [23,24]. It can also be seen from the results that the majority of participants believe that learning online assisted them to attend classes conveniently no matter where they were. This result is in keeping with the conclusions of Cook et al. [25], who established that learning online can boost learners’ perceived control over three elements: curriculum, locations, and timing/duration.

The present study’s findings are in keeping with the conclusions reached by Thompson [26] from a survey of 325 Saudi Arabian students. The study explored the effects of the COVID-19 pandemic on cognitive and behavioural engagement. Thompson reports that students perceived online learning as convenient but still believed that there was a decrease in educational quality, particularly with regard to the amount of knowledge they got. In Thompson’s survey, the students reported three concerns:

1. Cognitive disengagement, including a loss of enthusiasm in learning, challenges with focusing, and behavioural disengagement due to inability to see peers and professors.
2. Lecture delivery in online learning lacked standardisation, and students believe that structure and standardisation should be introduced so that lecturers follow a standard method of teaching.
3. Online assessments, which could have a negative impact on student grades. The students mostly complained about online tests.

The findings of the current study are also consistent with the results of other past studies, such as those conducted by Wang and Wang [27] and Chen and Tseng [28]. These studies also concluded that the effectiveness of online learning was positively impacted by online availability and connectivity. Thus, in this model, it is posited that online availability and connectivity form the prerequisites for an effective and acceptable online learning system. The present study found that a stable and strong internet connection and speed plays an important role in improving learning online because this facilitates the requisite connections with students and teachers. These are conclusions consistent with previous research indicating that online platforms have an important role to play by boosting interaction between people in the delivery of online lectures [29,30].

The majority of participants indicated that they found online classes interesting. The same conclusions were drawn by Ozaydın Ozkara and Cakir [31], who found that one of the advantages expressed by their participants was that the courses attended online were enjoyable. Nonetheless, this finding does not agree with the results of Williams and Pury [32], whose participants indicated that they did not find the online courses enjoyable.

With regard to the difficulties faced by students during online classes in the COVID-19 period, the results of the present study indicate that technical challenges were the major challenges confronted by students. The technical challenges identified in the current study can be divided into two categories. In the first category are technological issues such as the absence or lack of reliability of the internet or high-quality devices. The other category is linked to the learners as users, such as having a lack of technical skills, which can be improved through training and experience. The technical challenges noted in the current study are in keeping with the findings of several researchers [33,34,35].

Since all the participants in this study were female, it is important that the findings are interpreted with the views of Horvat et al. [36]. These researchers note that in online learning, female users of the system performed better compared to their male counterparts with regard to average waiting time for a response, material quantity, cooperation diversity, website user-friendliness, material clarity, material thoroughness, and feedback quality. A study conducted by Aesaert and Van Braak [37] also concluded that girls performed better than boys in internet technology skills.

Jæger and Blaabæk [38] reported that access to resources differed based on whether students came from low-income or high-income families. This is a disparity that exists across the globe, particularly in the developing world. Additionally, the coverage and speed of the internet differ depending on a students’ budget and location. For instance, students living in rural areas face challenges with regard to securing adequate internet access [39], in comparison to their peers residing in urban areas [40]. Therefore, in the event that classes and assessments are conducted fully online, some students may be treated unfairly. When combined with a lack of access to resources such as devices and the internet, students from low-income families may also be distracted and interrupted by family responsibilities [41].

The kingdom of Saudi Arabia is a monarchy with a constitution based on Islamic regulations [42], and, consequently, Islam permeates all aspects of life, including education [43]. Islam places special importance on education, and education is therefore a religious obligation. In Saudi Arabia, religion and education are inseparable; thus, the objective of education is not merely academic development, but also the development of Islamic religious consciousness [44]. Online learning is affected by cultural factors, for example, the presentation style of the curriculum, the individual educational style, and the relationship between teachers and students [45]. Although we cannot report directly relevant data, we anticipate that future research might profitably investigate the specific impact of Islamic values on online learning and education, more generally in Saudi Arabia.

### Limitations, Recommendations, and Implications

Online or distance learning does not only involve connection to the internet but also encompasses other elements such as blended technology training. In Saudi Arabia, there are many internet service providers. Therefore, to assist struggling families with secure internet access, authorities could work with service providers to make free or low-cost internet available.

Oproiu [46] conducted a study involving the electronic educational platform Moodle and concluded that the ability to connect via the internet makes the communication process between the teachers and students more effective. A study by Damnjanovic et al. [47] identified a number of factors, including system quality, satisfaction, perceived usefulness, performance outcome, information quality, and communication, as having a substantial effect on students’ decision to use Moodle.

There is a difference between students who choose to study online because they have selected distance education as a learning model and students who have been forced by a situation such as COVID-19 to study online. The latter may not be prepared psychologically and technologically for this type of learning. As a result, students indicated their apprehension with regard to the effect of such a situation on their performance. This calls for programs that take these concerns into account [48], including conducting additional research to measure the impact of migrating to online learning on the performance of students [49].

It is suggested that those semesters during which students were taught using online learning due to COVID-19 should be evaluated to determine the extent to which such learning was able to meet the standards as set by higher education authorities across the world. This is the first time that many students and lecturers faced online learning and associated challenges on such a massive scale. This is expected to have impacted the education process with regard to lectures, lab experiments, and assessments. Considering that education may have been substantially affected by changes [50] that could have long-term transformative effects [51], it is important for universities to prepare for a post-COVID-19 experience unlike that existing before the pandemic [49].

Vesely et al. [52] arrived at the conclusion that the increased interaction between students and faculty leads to greater satisfaction levels for online learners. On this basis, it is posited that more studies are required to adequately record these experiences and the changes that have been noted in teaching, learning, and assessment practices amid COVID-19 and the impact these changes have had on systems of education in varying contexts [53], including the Saudi Arabian and other Middle Eastern educational contexts.

## Figures and Tables

**Table 1:** Available demographic information for interviewees.

Name	Age (Years)	Level of Education
Noor	19	First year in humanities
Sarah	21	Fourth year in humanities
Nada	20	Second year in health science
Amal	20	Second year in humanities
Rahma	21	Third year in humanities
Hanan	20	Second year in general science
Rana	22	Fourth year in humanities
Sahar	21	Third year in humanities
Hesah	20	Second year in human rights
Jana	20	Second year in pharmacy

## Data Availability

Due to the nature of this research, participants did not agree to having their identified data be shared publicly.
